# Unsafe commute driving behaviour among healthcare workers: a combined scoping review and concept analysis

**DOI:** 10.2478/aiht-2025-76-3930

**Published:** 2025-09-30

**Authors:** Khairil Idham Ismail, Hanin Farhana Kamaruzaman, Mohd Faiz Ibrahim, Jonathan Michael Bryce, Rosnah Ismail, Hanizah Mohd Yusoff

**Affiliations:** Universiti Kebangsaan Malaysia, Faculty of Medicine, Department of Public Health Medicine, Kuala Lumpur, Malaysia; Ministry of Health Malaysia, Medical Development Division, Malaysian Health Technology Assessment Section, Putrajaya, Malaysia; University of Glasgow, Institute of Health and Wellbeing, Health Economics and Health Technology Assessment (HEHTA), Glasgow, United Kingdom; National Institutes of Health, Institute for Medical Research, Environmental Health Research Centre, Selangor, Malaysia; INTI International University, Nilai, Malaysia

**Keywords:** accidents, distraction, driver performance, fatigue, human factors, occupational health, risk factors, traffic, higijena rada, ljudski čimbenici, smetenost, promet, prometne nezgode, rizični čimbenici, vozačke sposobnosti, zamor

## Abstract

Unsafe driving behaviour is associated with the risk of crashes. Although commuting crashes prevail among healthcare workers (HCWs), unsafe driving behaviour during daily commutes remains unexplored in this group. The aim of our study was therefore to address this gap and to clarify the concept of unsafe driving behaviour among HCWs while commuting. To do that, we ran literature search in Medline, CINAHL, Scopus, and Web of Science and selected appropriate articles following the scoping review procedure, while data extraction and analysis followed the procedure for concept analysis. A total of 46 published studies met inclusion criteria. Most were from the USA (n=30), predominantly involved medical doctors (n=21), and were cross-sectional (n=24) in design. Concept analysis identified four properties of unsafe driving behaviour: 1) pressure and negative emotion, 2) drowsy driving, 3) risky driving and rules violation, and 4) distraction/inattention. Work scheduling factors emerged as the most frequently reported antecedents, while crashes were the most reported consequences. By identifying the core elements of unsafe driving behaviour among HCWs this study proposes a conceptual framework to guide future research and interventions. This framework can serve as a valuable resource for policymakers and researchers, enabling them to develop targeted strategies to address unsafe driving behaviour of HCWs during commuting, with the ultimate goal to reduce the associated crash risks.

## Abstract (hr)

Nesigurna vožnja povezana je s rizikom od prometne nezgode. Premda su nezgode učestale u zdravstvenih radnika tijekom vožnje na posao i s posla, njihovo vozačko ponašanje i dalje je slabo istraženo. Stoga je cilj ovog istraživanja bio utvrditi upravo takvo ponašanje u vožnji i razjasniti koncept nesigurne vožnje i rizičnih postupaka u zdravstvenih radnika. U tu smo svrhu pretražili dostupnu literaturu u indeksnim bazama *Medline*, *CINAHL*, *Scopus* i *Web of Science* te izabrali odgovarajuće članke slijedeći postupak za pretražni pregled, a za vađenje podataka i njihovu analizu postupak uobičajen za konceptualnu analizu. Ukupno je 46 objavljenih istraživanja zadovoljilo kriterije ulaska u analizu. Većina je iz SAD-a (n=30), uglavnom se odnose na liječnike (n=21) te su po dizajnu presječna (n=24). Konceptualnom analizom izdvojili smo četiri svojstva nesigurnog ponašanja u vožnji: (1) pritisak i negativne emocije, (2) vožnja u pospanom stanju, (3) rizična vožnja i nepoštivanje prometnih propisa te (4) smetenost/nepažnja. Najčešćim uzrocima pokazali su se čimbenici povezani s rasporedom i vremenom rada, a najčešće su posljedice bile prometne nezgode/sudari. Prepoznavši ključne elemente nesigurne vožnje među zdravstvenim radnicima, naše istraživanje nudi i konceptualni okvir za daljnja istraživanja i intervencije u ponašanju vozača. Taj okvir može poslužiti i kao vrijedan izvor informacija za zakonodavce i znanstvenike, omogućujući im da osmisle ciljane strategije rješavanja problema nesigurne vožnje tijekom odlaska na posao i povratka s posla kako bi se u konačnici smanjio rizik od prometnih nezgoda povezan s dnevnim migracijama zdravstvenih radnika.

Road traffic injuries account for over 1.35 million deaths worldwide each year, making them one of the leading causes of death. In addition, they account for about 50 million non-fatal injuries a year (1), which places an enormous burden on healthcare systems and strains the economic and social fabric of affected communities. A specific subset of incidents deserves special attention – commuting crashes – given that the average time travelled per 10 km has increased worldwide. In Bangkok, 26.15 % of residents commute one to two hours a day, 36.66 % in Istanbul, 32.72 % in Sao Paulo, and 28 % in New York (2, 3).

Among these commuters, healthcare workers (HCWs) bear a distinctive burden, and their experiences are particularly noteworthy. As they navigate their daily commutes to and from healthcare facilities, they confront a range of unique challenges, stemming from the urgency to reach their workplaces in time, irregular work hours, and the stressful environments in which they work (4,5,6,7). These factors combine to create a commuting experience that is exceptional and, in many ways, unmatched in its complexity and impact on driving behaviour. The incidence of commuting crashes among HCWs has increased by 15–18 % in France (8, 9) and Malaysia (10, 11), and HCWs have more road crashes than the rest of the population (25 % vs 12 %; p=0.002) (12). Trainee physicians working longer hours (>24 h) are more likely to be involved in road crashes (CI 1.6–3.3; p=0.001) (13). These factors make HCWs a population worthy of particular scrutiny and study.

Existing research has diligently identified traffic violations, errors, lapses, speeding, driving while tired, and inattention, as causes and contributing factors of road accidents (14, 15), yet the map remains largely uncharted, with a notable gap in our understanding of the distinct characteristics of unsafe driving behaviour during daily commutes, especially that of HCWs. Most research in this area has focused on the driving behaviours of the general population or specific groups such as shift workers or long-distance drivers (16, 17). Research in HCWs predominantly investigated those who drive as part of their job, such as emergency and home healthcare service drivers (4,5,6,7). The aim of this study was to identify the key properties of unsafe driving behaviour among commuting HCWs in order to better conceptualise and effectively address the issue.

## METHODS

We followed a methodology combining the scoping review and concept analysis reported by Lam et al. (18). This approach is wellsuited for identifying studies relying on diverse methodologies and evidence and is particularly useful for addressing the research question of what is known so far about the concept of HCWs’ unsafe driving behaviour while commuting. The approach consists of four main steps, each combining the scoping review and concept analysis: 1) selecting the central concept, excluding other related concepts, and setting the aim of the analysis; 2) identifying publications on the subject; 3) selecting relevant publications; and 4) charting and analysing the obtained data. The last step involves a descriptive quantitative summary of the obtained data [author(s), year of publication, first author country, study design, sample size, population characteristics, type of experimental studies (simulated vs naturalistic)] and concept analysis defining attributes, antecedents (causes), consequences (effects), and empirical referents of unsafe driving behaviour.

### Central concept, related concept, and aim of analysis

For our central study concept, we selected unsafe driving behaviour while commuting to and from work, as it deviates from responsible and safe driving practices and includes speeding (19, 20), rules violation (21,22,23), drowsy or fatigue driving (24,25,26), and distraction (27, 28).

Before we moved on, however, we had to exclude the related concept of on-duty, i.e., professional driving, to maintain focus on unsafe behaviours specific for commuting. Table 1 shows differences in characteristics between commuting and on-duty driving.

**Table 1:** Differences between commute and on-duty driving (central and related concept, respectively)

**Characteristics**	**Commute driving**	**On-duty driving**
Purpose of driving	To work and back home	For work and other purposes
Vehicle	Own vehicle	Employer’s vehicle
Passengers	Alone or with spouse or children	Alone or with colleague
Traffic hours	Usually peak hours	Throughout the day
Traffic congestion	High during peak hours	Subject to traffic hours
Road familiarity	Highly likely	Highly unlikely
GPS usage	Unlikely (except for traffic alerts)	Highly likely
Mode of driving	Highly “auto-mode”	More vigilant

In addition to the common characteristics of unsafe driving behaviours, commuting introduces several distinct contextual factors. These include time constraints and familiarity with routes, which together can significantly impact driving. Time pressure may lead to behaviours such as speeding, which increases the risk of accidents. Familiarity with everyday routes can foster complacency and an “auto-pilot” mode of driving, dulling the sense of alertness crucial for anticipating and responding to potential hazards. Furthermore, commuting often involves driving during peak traffic hours and higher congestion, which inherently increases the likelihood of accidents (29,30,31,32).

Moreover, other characteristics, while not exclusive to commuting, can profoundly affect HCWs’ driving behaviour while commuting due to their unique occupational demands. These include stress from work-related factors (e.g., emergency phone calls received while driving, or the psychological burden of work itself), frustration arising from traffic or work-related issues, and pervasive exhaustion from demanding shifts. Such factors can significantly contribute to distraction and inattention. As a result, personal communications and mental preoccupation often increase during commutes, potentially leading to more aggressive and therefore risky driving behaviours.

The second step was to identify relevant publications that fit our concept analysis parameters. To this end, we relied on the population, exposure, and outcome (PEO) mnemonic format (33, 34). We ran a systematic database search across Web of Science, Scopus, MEDLINE, and CINAHL by entering the following key words: “driv* behaviour”, “drowsy driv*”, “commuting” “risky driv*”, “unsafe driv*”, “healthcare workers”, and “nurs*” and by combining them with the Boolean operators ‘AND’ and ‘OR’. To ensure a systematic search, we applied the Preferred Reporting Items for Systematic Reviews and Meta-Analyses extension for Scoping Reviews (PRISMA-ScR) checklist (35). The search was limited to articles in English published between 1990 and 2022.

All studies from the initial search were exported into the CSV format and compiled into the EndNote X9 reference management software (Clarivate Analytics, Philadelphia, PA, USA) (36). The selection was then refined to meet the following inclusion criteria: HCWs of any discipline and level of profession, such as physicians, nurses, medics, paramedics, residents, health assistants, interns, and house officers (junior medical doctors in postgraduate training) and articles whose subject was driving behaviour. To expand our understanding of unsafe driving, we included papers that used driving simulators to assess driving impairment among HCWs.

The exclusion criteria were: non HCWs, articles mixing data on HCWs and other professions, articles with incomplete data on unsafe driving behaviour, and conference or editorial articles.

Where appropriate, data from the above databases were expanded to include other related studies identified through footnotes, citation tracking, and reference lists. Duplicate articles were removed manually. Titles and abstracts were screened by two independent reviewers and disagreements were resolved through consensus.

### Charting and analysing data

Numerical analysis was employed to summarise the frequency of the included studies by author(s), year of publication, first author country, study design, sample size, population characteristics, and type of experimental studies (naturalistic vs simulated) if applicable. In the context of driving behaviour research, these terms refer to distinct yet complementary approaches. Naturalistic studies involve observing driver behaviour in everyday real-world conditions using in-vehicle data recording equipment. Simulated studies utilise driving simulators in a controlled laboratory setting, allowing researchers to precisely manipulate environmental variables and test hazardous scenarios safely, while also facilitating precise measurements of driver performance and physiological responses. Both naturalistic and simulated studies help to understand driving behaviour.

Concept analysis was employed to identify the attributes of unsafe driving behaviour while commuting, as well as what preceded such behaviour (antecedents) and what the consequences were. In this analysis, the “event” under examination specifically refers to the manifestation or occurrence of unsafe driving behaviour. An antecedent is a factor, condition, or stimulus that precedes and is presumed to contribute to, initiate, or influence this unsafe driving behaviour (e.g., fatigue from a long shift, mental preoccupation, or time pressure). Conversely, a consequence is an outcome, result, or effect that follows directly from unsafe driving (e.g., a traffic collision, a near-miss incident, or a traffic violation). Additionally, we identified empirical referents, which serve as observable and measurable indicators of unsafe driving behaviour or its attributes (e.g., percentage of eyelid closure for drowsiness, recorded speeding for risky driving, or prolonged glances away from the road for distraction).

## RESULTS

**Figure 1:**
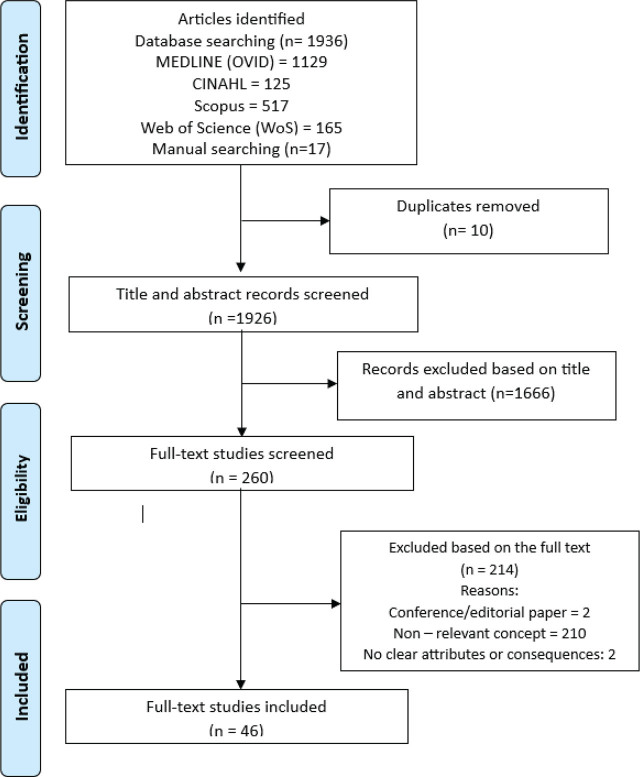
Article search strategy based on the PRISMA guidelines (35)

The four databases yielded 1949 articles in total. Upon screening, 46 were included in this review for further analysis (Figure 1). Most come from the USA and involve medical doctors and nurses. Their number increased between decades. Nearly half employed crosssectional design (n=21/46). Seven used driving simulators, four naturalistic observations, and the rest self-reported observations (Table 2).

**Figure 2:**
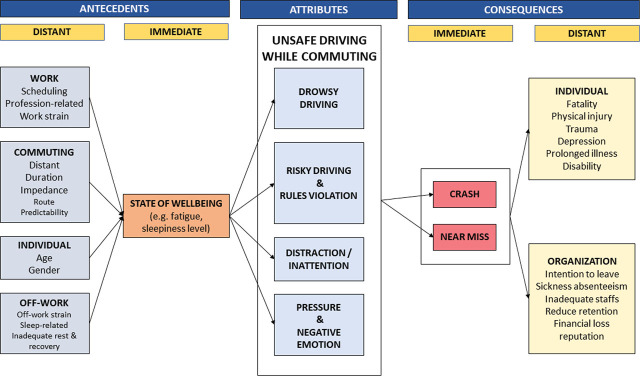
Factors affecting commute driving among HCWs

**Table 2:** Characteristics of the reviewed studies (n=46) on HCWs’ commute driving behaviour

**Type of data**	**Characteristics (no. of studies)**
Country	Australia &/New Zealand (n=6)
	Canada (n=1)
	France (n=1)
	Israel (n=2)
	Malaysia (n=2)
	Norway (n=1)
	United Kingdom (n=2)
	United States of America (n=30)
	Not stated (n=1)
Year of publication	1990–2000 (n=5)
	2001–2010 (n=10)
	2011–2020 (n=26)
	2021–2022 (n=5)
Study design	Cross-sectional (n=21)
	Case-control (n=2)
	Cohort/longitudinal (n=10)
	Quasi (pre/post) (n=7)
	Randomised clinical trial (n=2)
	Qualitative (n=4)
Study population	Doctors (including residents/interns/house officers) (n=24)
	Nurses (n=15)
	Mixed HCWs (n=7)
Experimental studies (N=11)	Driving simulator (n=7)
	Naturalistic observation (n=4)

### Results of concept analysis

We identified four main attributes in the following order of frequency (high to low): 1) being asleep at the wheel/drowsy driving (n=28); 2) rules violation (e.g., speeding, ticketing) (n=8); 3) inattention/distracted driving/rumination (n=7); and (4) stress/negative emotion (n=2) (Table 3).

**Table 3:** Summary of analysed studies by attributes of unsafe driving behaviour of HCW commuters and consequences of such behaviour

**Attributes**	**Number of studies**	**References**
Being asleep at the wheel / drowsy driving	28	(12, 13, 24, 37,38,39,40,41,42,43,44,45,46,47,48,49,50,51,52,53,54,55,56,57,58,59,60,61)
Rules violations (e.g., speeding, ticketing)	8	(12, 24, 53, 61,62,63,64,65)
Inattention / distracted driving / rumination	7	(24, 43, 52, 53, 61, 63, 665)
Stress / negative emotion	2	(55, 67)
**Immediate consequences**		
Near crash / hazardous event	21	(13, 24, 37, 46,47,48, 52,53,54, 56, 57, 61, 62, 64, 68,69,70,71, 75, 77, 78,)
Crashes / collisions	24	(9,10,11,12,13, 37, 39, 40, 42,43,44, 46,47,48,49,50, 53, 59, 61, 64, 69, 70, 75, 77,)
**Distant consequences**		
Individual (physical injury, emotional injury, poor quality of life, depression, burnout, fatigue)	5	(9, 11, 37, 44, 68)
Organisational (sick absenteeism, lower work performance and enjoyment, intention to work fewer hours)	4	(9, 37, 41, 44)

Drowsy driving was identified by 28 studies through key terms such as drowsy driving, asleep at the stop, asleep at the wheel, behavioural microsleep, sleepiness while driving, dozing off, nodding off, drifting between lanes, near crashes, and difficulty gauging the speed and distance from other vehicles (12, 13, 24, 37,38,39,40,41,42,43,44,45,46,47,48,49,50,51,52,53,54,55,56,57,58,59,60,61). While most studies utilised self-reporting, three utilised blinking duration measured in a real-life setting as a proxy of drowsy driving (52,53,54). Studies analysing road crashes indicate that drowsy driving accounts for 30–84 % of them (40, 44, 47,48,49).

Eight studies reported risky driving and rules violations based on self-reported behaviour described with the following key terms: traffic citations for moving violations, speeding, running through stop lights, driving events, and extreme speeding (12, 24, 53, 61,62,63,64,65). Among the studies reviewed, a case-control study (12) reported significantly more traffic citations issued to house officers than non-house officers (25 % vs. 18 %, p=0.001). Supporting these real-world findings, driving simulator studies provide controlled insights into job-related impairment. For instance, a driving simulator study involving 32 residents (junior medical doctors) found that these individuals drove significantly faster post-duty (p=0.001) than pre- or off-duty (62).

Seven studies (24, 43, 52, 53, 61, 63, 66) identified inattention and distraction as attributes of unsafe driving behaviour while commuting with key terms such as inattention, lack of awareness, distraction, missed turn, inattention-related events, forgetfulness, mind-wandering, not stopping at red lights, not taking green lights, missing the exit to go home, not being alert, not noticing, drinking beverages, using a cell phone, checking a pager, personal care activities, or changing clothing. Simulator studies identified slower braking reaction time due to inattention/distraction (66).

Finally, stress and negative emotions were identified by two studies (55, 67) with the following key terms: anxiety, feelings of stress, and outwardly expressed anger or frustration (e.g., shouting or driving angrily). These emotional states were often associated with behaviours such as reckless driving and consequences such as traffic citations.

Table 4 and Figure 2 break down the antecedents to unsafe driving behaviour while commuting into immediate and distant. The immediate antecedent is the individual’s state-of-wellbeing (n=15/46) described by key terms such as fatigue, exhaustion, sleepiness, tiredness, emotional exhaustion, inadequate recovery, poor psychosocial wellbeing, and lack of personal accomplishment.

**Table 4:** Antecedents influencing unsafe driving behaviours while commuting among HCWs according to themes, sub-themes, and keywords

**Groups**	**Antecedents**	**Key terms**	**References**
Work	Scheduling	Night shift, long shift, quick return (<11 h between shifts), type of shift, extended duration of shift, on-call, post duty	(9, 13, 32,33,34, 36, 40, 41, 44, 45, 47, 49,50,51,52, 54,55,56,57,58,59,60,61,62,63,64,65,66)
	Profession-related	Residency years, seniority, type of profession (doctor vs nurse vs paramedic, etc.), seniority, medical discipline (critical care vs emergency)	(9,10,11, 38, 39, 42, 44, 67)
	Pressure at work	Stress level, burnout (emotional exhaustion, personal accomplishment, depersonalisation), depression	(50, 68, 69)
Commuting	Commuting	Commuting distance, commuting duration, commuting impedance, rural vs urban, time spent actively driving, highlighting inherent task demands, direction of commuting (home to work vs work to home)	(9, 10, 35, 42, 48, 51, 60, 65, 70,71,72)
Off-work	Pressure outside work	Off-work stress, off-work activities (care for ageing parents, continuing studies while working, second job)	(69, 72)
	Sleep-related	Sleep duration, sleep quality, sleep deprivation, acute sleep loss, shift work sleep disorder (shift work difficulties, difficulties remaining awake at work, tendency to fall asleep, struggle to remain awake)	(12, 33, 37, 44, 47, 49, 50, 57, 58, 64, 67, 72)
Individual	Individual	Age (younger), gender (male),	(11, 38, 39, 67)
State of wellbeing	State of wellbeing	Fatigue, sleepiness, tiredness, mean blink duration (proxy for fatigue), percentage of eye closure (proxy for fatigue)	(32, 36, 39, 41, 45, 46, 49, 52, 58, 62, 67, 68, 72, 73)

Distant antecedents are divided into four categories as follows: 1) work-related, 2) off-work-related, 3) commuting, and 4) individual antecedents (Table 4). Work-related antecedents include scheduling issues (long working hours, irregular shifts, consecutive shifts, inadequate rest between shifts) (n=26/46), professional issues (8/46), and work strain (e.g., chronic job demands and high patient care responsibilities) (n=3/46). Off-work antecedents include offwork strain (e.g., heavy family responsibilities, financial difficulties, personal health challenges) (n=2/46), and sleep-related issues (e.g., insufficient total sleep duration, poor sleep quality, fragmented sleep due to shift work, sleep disorders) (n=12/46). Commuting antecedents are represented by commuting exposure (e.g., quantifiable aspects such as commute duration, distance, frequency, or traffic density) (n=11/46), and individual antecedents include age and gender (n=4/46). All distant antecedents to unsafe driving are pertinent to the immediate antecedent, that is, individual state of wellbeing (Figure 2).

Similarly, the consequences of unsafe driving while commuting are divided into immediate and distant. Immediate consequences include key terms such as near miss, hazardous event, or near crash (n=21) or actual road crash, collision, or accident (n=24). Distant consequences are divided into individual and organisational. Individual consequences include key terms such as fatality, physical injury, emotional injury, poor quality of life, depression, burnout, and fatigue, whereas organisational consequences involve sick absenteeism, reduced work performance, diminished work enjoyment, and intent to work fewer hours following the completion of residential training.

#### Empirical referents

The most often reported unsafe driving behaviour attribute was drowsy driving / asleep at the wheel. In self-reporting studies it was measured by a single item, such as “*do you feel drowsy while driving home?*”, or “*did you experience an accident or near-accident while driving home from work today?*” on the linear analogue self-assessment (LASA) scale for fatigue (68), the Karolinska Sleepiness Scale (KSS), and the Epworth Sleepiness Scale (ESS) before, during, and after driving (24, 52, 66).

A single item construct may be of limited accuracy and reliability and does not capture all the nuances of a concept. Moreover, it may be subject to bias due to response distortion. However, single-item constructs have the advantage of being quick and easy to administer and can prove useful in situations where multiple items are not feasible, such as in real-time settings in which some constructs are difficult to measure.

Several studies have measured fatigue or drowsy state objectively, using infrared oculography, average blink duration, and total blink duration (24, 52,53,54). In driving simulator and naturalistic observation studies, variables like steering variability, lane deviation, lane variability and slow braking reaction time were used as proxies for poor driving performance (62,63,64, 66, 69,70,71). Other driving simulator metrics included speed, speed variability, crashes, excessive speeding, weaving in and out of lanes, and delayed emergency braking reaction times. These correspond to hard brakes, swerves and near crashes in naturalistic observation studies.

## DISCUSSION

### Evolution of concept analysis for unsafe driving

The combined approach of a scoping review and concept analysis provides a more comprehensive understanding of unsafe driving of HCW commuters and the evolution of this concept in the literature, which has evolved significantly over the past three decades. Initially, the focus was on the incidence of near and actual crashes but turned out to be insufficient and gradually shifted towards preventable risk factors to encompass more nuanced behaviours such as speeding, reckless driving, using social media, answering phone calls and texting while driving, thinking about work while driving, distracted driving, and fatigue driving. All of these behaviours have emerged in recent studies as attributes of drowsy driving, recklessness, rule violation, and inattention (12, 24, 49, 52, 56).

Drowsy driving was identified by the majority of studies, as expected, considering that HCWs often work night shifts (which can disrupt circadian rhythm) (72) and long hours (which can lead to sleep deprivation) (64, 73). Meanwhile, feeling pressure and negative emotions were the least frequently reported attributes. These are consistent with the construct of the Dula Dangerous Driving Index (DDDI), which measures self-reported frequency of driving recklessly in response to feeling angry, rushed, or stressed (74).

New factors have also been identified. While early studies focused on the effects of scheduling such as night shifts and long work hours on safe driving (12, 39, 75), more recent studies identified other contributing factors such as those occurring outside work hours, during the commute, and individual factors. In that sense, qualitative studies provide a more comprehensive understanding of unsafe driving while commuting (55,56,57,58) as they address indirect factors, such as work and life imbalance, off-work pressure, stress, and recovery opportunities (e.g., adequate rest and sleep, leisure, personal stress-reducing activities, sense of accomplishment) (49). These factors may not be easily quantifiable but can still greatly affect driver safety.

Our review also shows that the main factors investigated by studies from 1990 to 2010 were work schedules (long hours, on-call duty, night shift, and sleep deprivation (13, 37, 39, 50, 51, 62, 69, 75, 76). While these continue to be studied, other factors have emerged as potential threats over the last two decades, including fatigue, workload, commuting distance, and commuting impedance (9,10,11, 40, 42, 44, 52, 53, 63, 77).

### Evolution of study design to analyse the concept of unsafe driving behaviour among HCWs

To better understand the role of antecedents in unsafe driving behaviour, various study designs have been used beside the cross-sectional (7, 37, 40, 41, 43, 44, 75, 78), such as diary (24, 50, 51), cohort (13, 68, 76), simulator and actual vehicle driving (naturalistic observation) (52, 54, 62,63,64, 66, 69,70,71, 79), some of them employing objective indirect measurements of drowsy driving (e.g., percentage of eyelid closure, blink duration, or saccadic eye movements) (52,53,54).

Interventional studies have also been used to address the issues of unsafe driving behaviour. The first such studies were focused on scheduling factors, but recent research has moved on to policies such as ride sharing (77), napping opportunities (49), fatigue mitigation (59), defensive driving techniques, stress management, and technology to reduce distractions while driving (56).

Some improvements have also been introduced to study design and methods. For instance, researchers have begun to use mixed-method, intensive longitudinal (24, 52, 53), naturalistic observation, and randomised controlled trial designs (71, 79). They have also introduced new objective measurements such as cortisol levels, heart rate variability, percentage of eye closure (52, 54), actigraphy, and driving simulators to measure physiological, psychological, and behavioural responses (63, 64, 69). This has led to more accurate and reliable results.

### Theoretical considerations

Most studies included in this analysis lack a theoretical explanation for unsafe driving behaviour while commuting, but there are several theories that may help understand the concept at hand.

Hockey’s cognitive-energetic model (CEM) can explain how cognitive and energetic factors interact to influence driving behaviour (80). It highlights various behaviours that can occur when goal, motivation, resources, and task performance are confronted with external loads such as stress or fatigue. In the commute driving context, these behaviours include yawning and nodding off at a red light, angry driving, drifting into the wrong lane, and having trouble steering. In addition to behavioural responses, there are individual psychophysiological responses such as increased heart rate, respiration, sweating, and muscle tension triggered by the body’s autonomic nervous system when an individual perceives a threat.

The generic error-modelling system (GEMS) (15) focuses on human factors that contribute to unsafe driving like violations, errors, and lapses, examples of which include speeding, illegal U-turns, poor judgment, rushed thinking, or inattention to the road.

The third is Heinrich’s domino theory (81), which postulates that accidents are caused by a series of factors or events which resemble dominoes that fall in a chain reaction. The first domino includes undesirable elements from both nature and nurture. The following dominos include undesirable factors from work, outside work, during commuting, and individual factors.

### Recommendations for future studies and policies

Our study has identified several gaps in the literature it encompassed. The first is a lack of standardised definitions and measures for unsafe driving behaviour among HCWs while commuting, which makes it difficult to compare and synthesise findings across studies. The second is that the literature rarely discusses what is unique about commuting in terms of its characteristics, such as familiarity with the route, auto-mode driving, and complacency. The third is that most studies cover commuting in developed countries. The fourth is that they are predominantly cross-sectional and rely on self-reported data, which may be subject to bias and inaccuracies.

More research is needed on other unsafe driving behaviours besides drowsy driving when commuting, such as on angry and resentful driving and complacency owed to familiarity with the route. While some studies have identified factors such as fatigue and time pressure, there is a need for more in-depth exploration of the underlying cognitive and psychological mechanisms that lead to unsafe driving behaviour in this population.

Lastly, only a handful of studies were specifically designed to test interventions aimed at preventing drowsy driving and road crash involvement, which calls for more targeted and tailored interventions that take into account the unique characteristics and demands of HCW commute driving. They may include interventions that reduce fatigue, enhance recovery, inform participants of safe driving and ride sharing practices, and address broader organisational and cultural factors that influence driving behaviour.

### Study strengths and limitations

The key strength of this study is that it combines scoping review of the literature and concept analysis, as this approach provides a comprehensive overview and systematic and rigorous analysis of existing literature on the topic. We have also suggested the use of theoretical models such as CEM, GEMS, and Heinrich’s domino to gain a deeper understanding of the factors contributing to unsafe driving among HCWs while commuting.

However, our study also has several limitations. Our focus on only English language sources may have excluded relevant studies and information from non-English speaking countries. Another limitation is the use of different methods for data analysis, as it may lead to discrepancies or inconsistencies in the findings. Furthermore, while providing a comprehensive overview of a broad spectrum of studies, their diverse design presents a challenge for direct comparison and quantitative synthesis due to inherent differences in their methodological strengths and the level of evidence they provide. This necessitates a careful qualitative interpretation of the findings. Finally, our study is limited by the quality and scope of the existing literature. Particularly if the topic is one where there are significant gaps in research.

## CONCLUSION

The increasing number of commuting accidents among HCWs raises concern about unsafe driving behaviours in this population group. Most analysed studies were conducted in the USA, mostly among doctors, and were cross-sectional in design. Our analysis has singled out drowsy driving, traffic violations, distractions, and negative feelings as the reported attributes for unsafe driving behaviours while commuting. HCWs seem to be more likely to engage in unsafe driving behaviours due to poor wellbeing factors related to work, time spent outside work, commuting, and individual characteristics.

Future research should expand on unsafe driving behaviours relevant to HCWs and commuting, including speeding, tailgating, time pressure-related traffic violations, and driving complacency owed to familiarity with the route.

Our approach to the concept may inform the development of a new scale to evaluate unsafe driving behaviours, identify potential issues, and provide actionable guidance for HCWs, their employers, and policy makers. Furthermore, this research can be expanded to other professions that require frequent commuting.
